# Clinical Characteristics of Paediatric Pancreatitis Caused by Pancreaticobiliary Malformation: A Single-Centre Retrospective Analysis

**DOI:** 10.3389/fped.2021.677894

**Published:** 2021-06-10

**Authors:** Jing Guo, Qian-ru Jia, Mei Sun

**Affiliations:** Department of Pediatrics, Shengjing Hospital of China Medical University, Shenyang, China

**Keywords:** pancreaticobiliary malformation, pancreaticobiliary maljunction, children, pancreatitis, magnetic resonance cholangiopancreatography

## Abstract

**Background/Aims:** To investigate the clinical profiles of children with pancreatitis caused by pancreaticobiliary malformation.

**Methods:** We retrospectively analysed the clinical data of children diagnosed with pancreatitis at our institute from June 2017 to January 2021.

**Results:** A total of 195 patients and 169 control subjects were included in this study. Twenty-six (13.3%) patients had pancreaticobiliary malformation-related pancreatitis. The average age of onset in the pancreaticobiliary malformation pancreatitis (PMP) group was lower than that in the non-PMP group, and the difference was statistically significant. The number of patients in the PMP group that had jaundice was significantly higher than that of the non-PMP group (*P* < 0.05). Logistic regression analysis showed that total bilirubin (TB) and γ-glutamyltransferase (GGT) (odds ratio = 1.096, *P* < 0.01) were independent predictors of pancreaticobiliary malformation-related pancreatitis in children. The positive detection rate of pancreaticobiliary malformation was 68% for abdominal ultrasound, 38.4% for abdominal enhanced computed tomography, and 91.3% for magnetic resonance cholangiopancreatography (MRCP). The recurrence rate (34.6%) in the PMP group was higher than that in the non-PMP group (15.4%, *P* < 0.05); surgical therapy had the lowest recurrence rate. Age at initial onset of pancreatitis was younger and the period to recurrence was shorter in the PMP group than in the non-PMP group (*P* < 0.05).

**Conclusion:** Pancreaticobiliary malformation is one of the major causes of paediatric pancreatitis. Elevated TB and GGT in patients with pancreatitis may be suggestive for underlying pancreaticobiliary malformation not solely to pancreatitis. MRCP should be used when pancreatitis due to pancreaticobiliary malformation is suspected. Surgery or endoscopic retrograde cholangiopancreatography-guided intervention may be helpful but further study is needed.

## Introduction

The aetiology of pancreatitis in children is complex and includes infection, metabolic disorders, drug use, biliary-related factors, trauma, the presence of systemic diseases, and genetic cause. Although numerous studies have focused on pancreatitis (1), few of those studies were on pancreatitis caused by pancreaticobiliary malformation. Pancreaticobiliary malformation combined with pancreatitis has atypical symptoms; thus, the diagnosis and treatment of the condition are often delayed, resulting in lower quality of life, and poor prognosis. The purpose of this study was to clarify the clinical characteristics of paediatric pancreatitis caused by pancreaticobiliary malformation pancreatitis (PMP) and non-PMP and to assess the differences between their recurrence rates to improve the detection rate of pancreaticobiliary malformation in children.

## Methods

### Patients

In this single-centre retrospective study, we enrolled a total of 195 children diagnosed with pancreatitis and treated at our institute between June 2017 and January 2021. Twenty-six children with pancreatitis due to pancreaticobiliary malformation were defined as the PMP group, whereas 169 children with no pancreaticobiliary malformation were defined as the non-PMP group.

### Inclusion Criteria

Children under 14 years old (because the paediatric department of our hospital only accepted children younger than 14 years old) who were diagnosed with any of the following: acute pancreatitis (AP) (2); chronic pancreatitis (2); acute recurrent pancreatitis (APP) (3); and pancreaticobiliary malformation, which included anatomical abnormality of the pancreaticobiliary duct system, including pancreas divisum, PBM (4), congenital cystic bile duct dilatation, and pancreatic duct strictures (5).

Definitions of AP, ARP, and CP were according to INSPPIRE Criteria (2, 3). AP requires ≥2 of the following 3 criteria: ① Abdominal pain symptoms consistent with AP (children under 3 years old or critically severe children may not have abdominal pain); ② serum amylase or lipase level ≥3 times greater than the upper limit of normal; ③ Imaging findings were in line with the imaging characteristics of AP. CP requires ≥1 of the following 3 criteria: ① Abdominal pain and imaging examination showed characteristic changes of CP; ② Insufficient pancreatic exocrine function and imaging examination showed characteristic changes of CP; ③ Insufficient pancreatic endocrine function and characteristic changes in imaging examination. ARP requires ≥2 episodes of AP plus complete resolution of pain (≥1-mo pain-free interval between diagnoses of AP) or complete normalisation of amylase and lipase between episodes.

### Exclusion Criteria

Simple choledochal cyst or bile duct malformation without pancreatitis.

### Clinical Information

Records of each patient's general condition, sex, age at the time of onset, clinical manifestations, body mass index (BMI), and recurrence were collated and analysed. Information on the levels of laboratory indicators including serum amylase (AMY), serum lipase (LPS), liver function indicators [alanine aminotransferase (ALT), aspartate aminotransferase (AST), total bilirubin (TB), γ-glutamyltransferase (GGT), total bile acid (TBA)], procalcitonin (PCT), interleukin 6 (IL-6), C-reactive protein (CRP), and haematocrit (HCT) were recorded. Fasting insulin and fasting blood glucose levels were recorded as well. The imaging examinations conducted included abdominal ultrasonography, computed tomography (CT), and magnetic resonance cholangiopancreatography (MRCP).

### Statistical Analysis

SPSS17.0 (SPSS Inc., Chicago, IL) was used for data analysis. Measurement data were expressed as mean ± standard deviation ($\bar{\text{X}}$ ± S). The *t-test* was used for comparison between groups, whereas chi-squared analysis was used for count data. *P* < 0.05 was considered statistically significant. After screening the factors with *P* < 0.05, multiple logistic regression analysis was carried out to evaluate the factors related to PMP in children.

## Results

### Differences Between the Clinical Profiles and Laboratory Results of Patients With PMP and Those With non-PMP

The comparison of the clinical profiles of the patients is shown in Table 1. A total of 195 children were included: 26 in the PMP group and 169 in the non-PMP group. Patients with pancreaticobiliary malformation accounted for 13.3% of the study population. The PMP group consisted of 9 males and 17 (65.4%) females; the male-to-female ratio was 1:1.89 and the average age of onset was 4.4 ± 2.73 years old. In the non-PMP group, there were 86 males and 83 (49.1%) females; the male-to-female ratio was 1:0.96 and the average age of onset was 6.2 ± 4.69 years old. The average age of onset in the PMP group was lower than that in the non-PMP group, and the difference was statistically significant (*P* < 0.05).

**Table 1:** Differences in clinical profiles between the PMP and the non-PMP groups.

	**PMP group**	**Non-PMP Control group**	***P***
Number of cases	26	169	
Female:male	1.89:1	0.96:1	<0.05
Age at onset of initial pancreatitis (mean ± SD, years)	4.4 ± 2.73	6.2 ± 4.69	NS
Clinical manifestations			
Abdominal pain (%)	84.6%	69.8%	NS
Vomiting (%)	76.9%	54.4%	NS
Fever (%)	23.1%	40.2%	NS
Jaundice (%)	7.7%	0	<0.01
BMI	15.07 ± 0.98	18.6 ± 5.14	<0.05

*BMI, body mass index*.

The main clinical manifestations of the 26 children in the PMP group were abdominal pain (84.6%), vomiting (76.9%), fever (23.1%), jaundice (7.7%), and upper abdominal tenderness (92.3%). The main clinical features of the 169 children in the non-PMP group were acute attack and persistent abdominal pain (69.8%) accompanied by vomiting (54.4%), abdominal tenderness (88.8%), and fever (40.2%). The number of patients with jaundice in the PMP group was significantly higher than that in the non-PMP group (*P* < 0.05). However, there were no statistically significant differences between the number of cases of abdominal pain, vomiting, and fever recorded for the two groups.

To evaluate whether obesity was an interference factor between the two groups, we analysed the weight, height, and fasting insulin and fasting blood glucose levels of the patients and calculated their BMI. As shown in Table 1, the BMI of the patients in the non-PMP group was significantly higher than that of patients in the PMP group (*P* < 0.05).

The comparison of the levels of the laboratory indicators for both groups is shown in Table 2. The ALT, AST, GGT, and TB levels of the patients in the PMP group were significantly higher than those of patients in the non-PMP group (*P* < 0.01). The HCT level of the PMP group was lower than that of the non-PMP group (*P* < 0.05). AMY, LPS, and TBA levels were not significantly different between the two groups (*P* > 0.05). There were no statistically differences between the PCT, CRP, and IL-6 in the two groups.

**Table 2:** Differences in laboratory indicators between the PMP and the non-PMP groups.

	**PMP group**	**non-PMP group**	**T**	***P***
AMY (U/L)	876.6 ± 229.0	397.5 ± 835.0	−1.052	NS
LPS (U/L)	1145.0 ± 985.4	714.4 ± 107.6	−1.851	NS
ALT (U/L)	114.1 ± 139.2	33.5 ± 60.5	−2.906	<0.01
AST (U/L)	107.2 ± 116.5	38.3 ± 39.6	−2.987	<0.01
GGT (U/L)	215.8 ± 299.5	25.5 ± 46.3	−3.233	<0.01
TB (μmol/L)	25.2 ± 33.5	8.6 ± 6.1	−4.739	<0.05
TBA (μmol/L)	10.0 ± 23.0	8.7 ± 56.5	−0.098	NS
PCT	0.25 ± 0.25	0.56 ± 1.92	0.642	NS
CRP (mg/L)	21.44 ± 32.37	23.18 ± 43.72	0.19	NS
IL-6	13.55 ± 19.05	23.71 ± 27.53	1.011	NS
HCT	36.4 ± 3.05	38.4 ± 4.56	2.018	<0.05

*AMY, serum amylase; LPS, serum lipase; ALT, alanine aminotransferase; AST, aspartate aminotransferase; TB, total bilirubin; GGT, γ-glutamyltransferase; TBA, total bile acid; PCT, procalcitonin; IL-6, interleukin 6; CRP, C-reactive protein; HCT, haematocrit*.

### Logistic Regression Analysis of PMP

The six indicators that showed statistical significance were tested for collinearity; the tolerance was >0.5 for all the indicators and no multicollinearity was noted. The statistical significance of each of the six indicators was tested for collinearity as well; the tolerance was >0.5 for all the indicators and no multicollinearity was noted. A multiple logistic regression analysis using the backward stepwise method, with the six indicators as independent variables and the observation and non-PMP groups as dependent variables, the result showed that TB (OR = 1.108, *P* < 0.01) and GGT (OR = 1.009, *P* < 0.05) were independent predictors of pancreaticobiliary malformation- related pancreatitis in children (Table 3).

**Table 3:** Logistic regression analysis on correlative factors of PMP in children.

**Factors**	**B**	**SE**	**Wals**	**OR**	**95%CI**	***P***
Age	−0.11	0.092	1.433	0.896	0.748~1.073	0.231
ALT	−0.009	0.006	1.970	0.991	0.979~1.003	0.160
AST	0.011	0.008	1.910	1.011	0.995~1.027	0.167
GGT	0.009	0.004	4.391	1.009	1.001~1.018	0.036
TB	0.103	0.035	8.712	1.108	1.035~1.187	0.003
HCT	−0.066	0.057	1.323	0.936	0.837~1.047	0.936

### Imaging Manifestations of PMP and non-PMP

**Figure 1:**
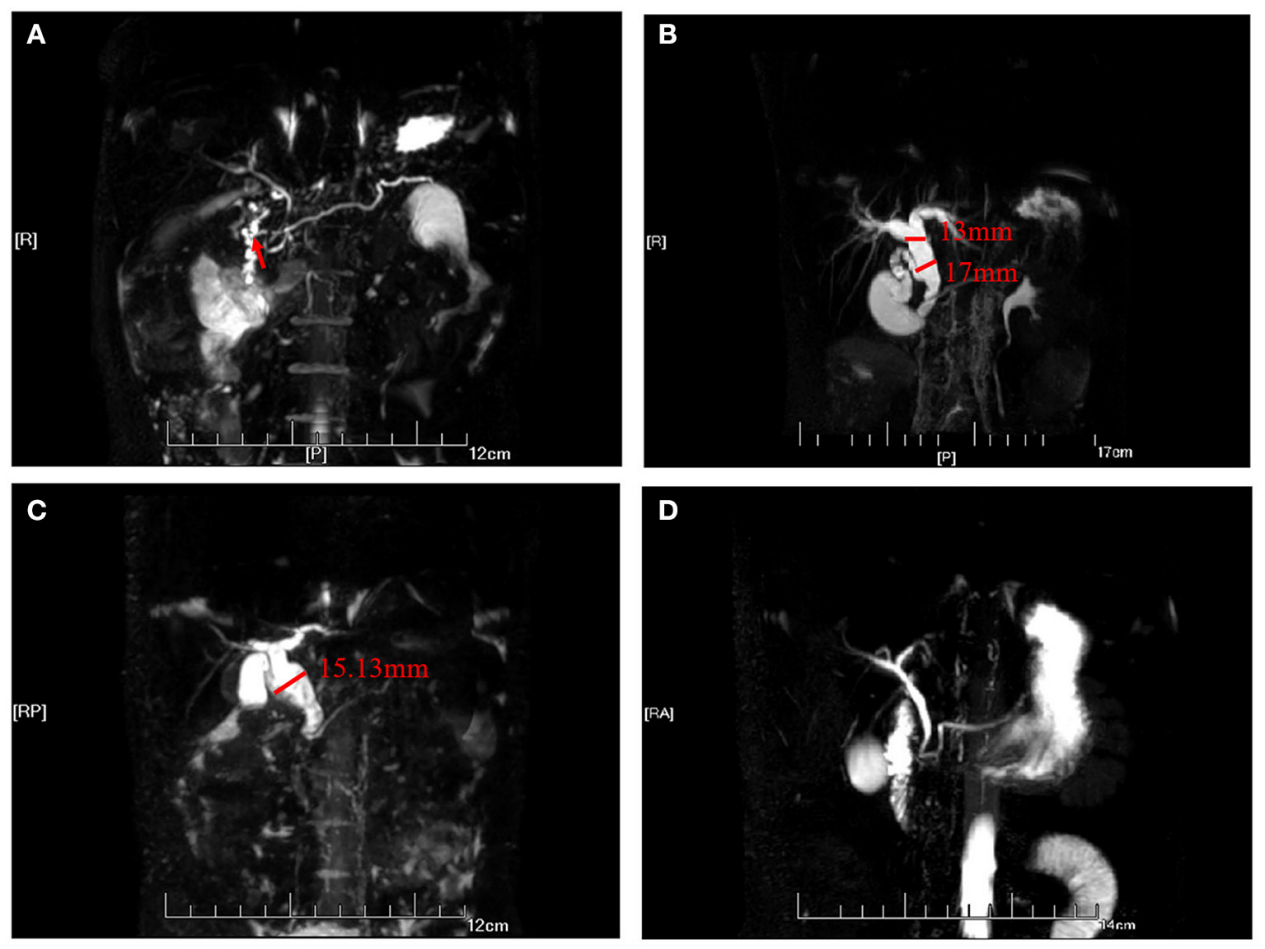
MRCP diagram of pancreaticobiliary duct dysplasia. **(A)** The main pancreatic duct was slightly dilated, stiffed, and merged into the minor duodenal nipple. **(B)** The intrahepatic bile duct, common hepatic duct, and common bile duct were widened. The widest part of the common hepatic duct had a diameter of 1.3 cm, and the diameter of the common bile duct was 1.7 cm. The opening of the cystic duct confluence was a lower, and the confluence of the common bile duct and pancreatic duct was locally fusiform dilation. **(C)** The common bile duct was dilated, with a diameter of 15.13 cm at its widest point. **(D)** The pancreatic ducts were widened, and the pancreaticobiliary ducts converged in advance. The length of the common duct was about 1.3 cm.

In the PMP group, the positive detection rate of pancreaticobiliary malformation with abdominal ultrasound was 17/25 (68%), that with abdominal enhanced CT was 5/13 (38.4%), and that with MRCP was 21/23 (91.3%). The pancreaticobiliary malformation include two cases of PBM, four cases of congenital type IA and one case of congenital type IC cystic bile duct dilatation combined with PBM, two cases of congenital type III cystic bile duct dilatation, 11 cases of congenital type IVA bile duct cystic dilatation combined with PBM, one case of congenital type V cystic bile duct dilatation, two case of congenital variation of pancreatic duct, and three cases of pancreatic duct strictures. Pancreaticobiliary malformation was accompanied by choledocholithiasis in 13 cases, gallbladder stones in 6 cases, and low biliary obstruction in 3 cases. Typical MRCP images are shown in Figure 1.

In the non-PMP group, 86 cases were diagnosed as pancreatitis by CT examination, 144 cases were diagnosed by abdominal ultrasound examination, and 55 cases were diagnosed by MRCP examination.

The positive detection rate of CT for pancreaticobiliary malformation was 5/(13+86) (5.1%), abdominal ultrasound was 17/(144+25) (10.1%), MRCP was 21/(55+23) (26.9%).

### The Treatment of PMP and the Differences Between the Recurrence Rates of the Two Groups

The basic treatments administered to the patients in the two groups were fasting, fluid resuscitation, enteral nutrition, administration of proton pump inhibitors, and other symptomatic therapy.

In the PMP group, seven patients underwent conservative treatment; 12 patients underwent surgical treatment, including choledochojejunostomy, Kasai procedure, pancreaticojejunostomy, percutaneous transhepatic cholangic drainage, cholecystectomy, and end-to-side jejunojejunostomy; and seven patients underwent endoscopic retrograde cholangiopancreatography (ERCP) interventional therapy, including endoscopic sphincterotomy, stenting drainage and bile ductal stone extraction.

As shown in Table 4, the recurrence rate (9/26, 34.6%) in the PMP group was significantly higher than that in the non-PMP group (26/169, 15.4%), *P* < 0.05. Recurrence frequency (3.66 ± 3.64) times was also higher in the PMP group than in the non-PMP group (1.3 ± 0.47) times, but there was no statistically significant difference between the two groups. The age at initial onset of pancreatitis was younger for patients in the PMP group (3.85 ± 3.34) years old than for those in the non-PMP group (6.68 ± 3.62 years old, *P* < 0.05). The interval of recurrence was 7.7 ± 8.52 months for the PMP group, which was shorter than that of the non-PMP group (15.44 ± 16.98 months, *P* < 0.05).

**Table 4:** Differences in clinical recurrence profiles between the PMP and the non-PMP groups.

**Recurrence**	**PMP group**	**non-PMP group**	***P***
Age at onset of initial pancreatitis (mean ± SD, years)	3.85 ± 3.34	6.68 ± 3.62	<0.05
Sex (male: female)	2:1	8:5	NS
Recurrence rate	9/26 (34.6%)	26/154 (15.4%)	<0.05
Period to recurrence, months (mean ± SD)	7.7 ± 8.52	15.44 ± 16.98	<0.05
**Recurrence cases**			
1 times	5	18	
2 times	0	8	
3 times	1	0	
≥4 times	3	0	
Mean ± SD	3.66 ± 3.64	1.3 ± 0.47	NS

Four of the seven patients who underwent conservative treatment had recurrences; the recurrence rate was 57.1%, and all of them had only one recurrence. Twelve children underwent surgical treatment, and one of them had only one recurrence (recurrence rate, 8.3%). The recurrence rate for the seven children who underwent ERCP-interventional treatment was 57.1%. Therefore, the recurrence rate for surgery was significantly lower than that for conservative treatment or ERCP-interventional treatment (*P* < 0.05). Of the five children who underwent ERCP-interventional treatment when pancreatitis was diagnosed for the first time, three did not relapse, and the two that relapsed were those who had pancreatic duct stenosis treated by ERCP stenting. Another child with pancreatic duct stenosis was treated with ERCP stenting after three relapses; however, the child still had one relapse after treatment. One child who was diagnosed with PBM and treated with ERCP stenting after nine recurrences still had recurring attacks afterwards; however, the child was finally treated with laparoscopic Roux-en-Y hepaticojejunostomy. In the four children who had recurrences ≥3 times, three children had pancreatic duct stenosis, and one had PBM.

In the non-PMP group, the imaging results of children with recurrence showed that 2 cases were gallbladder stones, 2 cases were pancreatic tumour, and 2 cases were pancreatic pseudocysts.

## Discussion

In this study, we evaluated the clinical characteristics of paediatric pancreatitis caused by pancreaticobiliary malformation and non- pancreaticobiliary malformation to improve the detection rate of pancreaticobiliary malformation in children. The aetiology of paediatric pancreatitis, which includes infections, systemic diseases, trauma, drug induction, biliary system diseases, anatomical abnormalities and genetic cause (*CFTR, PRSS1, SPINK1, CTRC* mutations), is significantly different from that of adults. Among these factors, congenital anatomical factors are responsible for a larger proportion of cases of paediatric pancreatitis. Early analysis of the cause and clear diagnosis are of great significance in the further treatment and improve the prognosis for children with this condition. This study is a retrospective study, so the limitations of the study is the inherit limitations in quality of data collected. Such as, only a very small number of recurrent pancreatitis completes genetic testing, so it is not counted in this article. And the genetic testing is important for the diagnosis of PBM, Guo et al. (6) reparted that *FUT1* and *MYBPC1* may be potential biomarkers for PBM.

Su et al. (7) reported that pancreaticobiliary structural anomalies account for 28% of cases of RAP. This study also indicated that the statistics of paediatric pancreatitis over the past 3 years show that pancreatitis caused by pancreaticobiliary malformation accounts for 13.3% of cases. This statistic confirms that pancreaticobiliary malformation is a very important cause of pancreatitis in children. Another study (8) showed that the structural pancreaticobiliary etiologic category accounts for 43.7% cases of pancreatitis among younger patients. In the present study, the average age of onset for the PMP group was significantly younger than that of the non-PMP group, reflecting the trend of younger age in the pancreaticobiliary malformation group, which is consistent with the report of the previous study. We also found that the ratio of girls in the pancreaticobiliary malformation group was higher than that of the non-PMP group. Thus, it can be considered that pancreaticobiliary malformation is more likely to occur in girls, a finding which is consistent with that of a previous report (9). Therefore, when younger children, especially girls, present with pancreatitis, the possibility of pancreaticobiliary malformation should be considered.

In the present study, the symptoms of children with PMP mainly included abdominal pain, vomiting, and fever; however, abdominal masses were not common. These symptoms are not very different from those of ordinary pancreatitis. Notably, compared with children with ordinary pancreatitis, few children with PMP had visible jaundice. Therefore, when children have pancreatitis with jaundice, it is necessary to pay attention to the possibility of pancreaticobiliary malformation.

The results of the present study also showed that the serum AMY and LPS levels of the patients in the PMP group were increased; however, there was no significant difference between the two groups in this regard. This result suggests that AMY and LPS cannot distinguish the cause of pancreatitis. This finding is consistent with those outlined in a previous report (10). The present study also showed that the ALT, AST, GGT, and TB levels of the patients in the PMP group were significantly higher than those of patients in the non-PMP group. This is considered to be related to obstructive jaundice and liver damage secondary to poor bile duct drainage. The study findings have similarities to a related publication by Coffey MJ (11). The levels of other inflammatory indicators such as PCT, CRP, and IL-6 were not significantly different between the two groups. It has been reported (8) that AST and obesity are independent predictors of biliary pancreatitis in children and that HCT, CRP and BMI are useful predictors of severe pancreatitis (12). However, the logistic regression analysis results of the present study suggested that TB and GGT are predictors of pancreatitis caused by pancreaticobiliary malformation. Therefore, children with pancreatitis who have elevated TB and GGT levels need to be pay more atthention to pancreaticobiliary malformation.

In the diagnosis of pancreaticobiliary duct dysplasia, the advantage of ultrasound (13) is that it is non-invasive and does not emit radiation. It can measure the diameter of choledochal cysts and detect cholecystitis and protein clots; however, an experienced ultrasound doctor is required to perform the procedure. CT (14) can distinguish liver cysts from intrahepatic and extrahepatic bile ducts and pancreatic ducts. It is also sensitive to the diagnosis of suspicious cancer (15); however, it has disadvantages in terms of measurement of common duct length and positive detection rate. MRCP (16) is a widely used non-invasive, low-risk technology. Compared with ultrasound and CT, MRCP can reflect the conditions of the intrahepatic and extrahepatic bile ducts, common bile ducts, and cystic ducts more clearly. It is more helpful in distinguishing PBM from congenital biliary dilatation (CBD) and in categorising CBD. MRCP examination has important clinical value in the early diagnosis of pancreaticobiliary dysplasia (17). The present study showed that MRCP has a higher positive detection rate and diagnostic significance for pancreaticobiliary malformation than other diagnostic methods previously mentioned. In summary, when children with pancreatitis have cholestasis or liver dysfunction, an MRCP should be considered to help facilitate diagnosis of underlying aetiologies.

It has been reported that patients with pancreaticobiliary duct dysplasia have an increased rate of malignancy as the disease progresses (18). For diagnosed children, surgical, or ERCP are recommended to reduce the risk of gastrointestinal cancer (19). The recurrence of AP is related to many factors (20), and the recurrence rate of pancreatitis caused by pancreaticobiliary malformation is up to 80% (21). The present study showed that the recurrence rate and recurrence frequency of pancreatitis caused by pancreaticobiliary malformation were higher than those of non-biliary pancreatitis. The results also showed that the surgical treatment of PMP had the highest remission rate and the lowest recurrence rate. We also noted that the recurrence rate of pancreatitis among children who underwent ERCP therapy after the first episode of pancreatitis was low. The treatment effect of pancreatic duct stricture was poor and the recurrence rate was the highest. This finding is inconsistent with that of a previous report (5) which showed temporary fully covered self-expandable metal stents placement was feasible and safe for the management of refractory benign main pancreatic duct stricture in children.This inconsistency may be related to the lower number of cases in the present study. For PBM children combined with choledochal cyst, excision of the extrahepatic bile duct, and hepaticojejunostomy are recommended (15). With the advancement of endoscopy technology, ERCP intervention is an effective and relatively safe treatment for PBM. Zeng JQ reported that the effective rate of ERCP therapy for PBM was 82.4%, the rate of RAP was 11.8% (22). Troendle et al. (23) suggest presence of ductal obstruction as a sign of likely clinical benefit with ERCP. Based on these findings, medical treatment for children with pancreatitis due to pancreaticobiliary malformation can only temporarily relieve the symptoms. Due to the small number of patients and the quite variable reasons of underlying pancreaticobiliary malformation, it is very difficult to extrapolate which patients received ERCP, which patients received surgery. So surgical or ERCP intervention may be helpful for PMP but further study is needed.

In summary, the clinical symptoms of children with PMP are typical to pancreatitis but not suggestive for underlying pancreatic malformation. This study showed that this type of pancreatitis has a high recurrence rate and is more common in young girls with clinical manifestations of jaundice. In addition to elevated LPS and AMY levels, attention should be paid to elevated ALT, AST, TB, and GGT levels in patients with pancreaticobiliary malformation, and elevated TB and GGT in patients with pancreatitis may be suggestive for underlying pancreaticobiliary malformation not solely to pancreatitis. MRCP has the highest detection rate for PMP. Surgery or ERCP intervention may be helpful for PMP but further study is needed.

## Data Availability Statement

The original contributions presented in the study are included in the article/supplementary material, further inquiries can be directed to the corresponding author/s.

## Ethics Statement

Ethical review and approval was not required for the study on human participants in accordance with the local legislation and institutional requirements. Written informed consent to participate in this study was provided by the participants' legal guardian/next of kin. Written informed consent was obtained from the individual(s) for the publication of any potentially identifiable images or data included in this article.

## Author Contributions

JG collected clinical data and wrote the article. QJ conducted the statistical analysis. MS was responsible for the revision of the manuscript to ensure the inclusion of important intellectual content. All authors approved the final draught of the paper for submission.

## Conflict of Interest

The authors declare that the research was conducted in the absence of any commercial or financial relationships that could be construed as a potential conflict of interest.
